# Continuous Low-Intensity Ultrasound Preserves Chondrogenesis of Mesenchymal Stromal Cells in the Presence of Cytokines by Inhibiting NFκB Activation

**DOI:** 10.3390/biom12030434

**Published:** 2022-03-11

**Authors:** Sarayu Bhogoju, Shahid Khan, Anuradha Subramanian

**Affiliations:** Department of Chemical and Materials Engineering, The University of Alabama in Huntsville, Huntsville, AL 35899, USA; sarayubhogoju@gmail.com (S.B.); sk0116@uah.edu (S.K.)

**Keywords:** mesenchymal stromal cells, ultrasound, NFκB pathway, mitochondrial potential

## Abstract

Proinflammatory joint environment, coupled with impeded chondrogenic differentiation of mesenchymal stromal cells (MSCs), led to inferior cartilage repair outcomes. Nuclear translocation of phosphorylated-NFκB downregulates SOX9 and hinders the chondrogenesis of MSCs. Strategies that minimize the deleterious effects of NFκB, while promoting MSC chondrogenesis, are of interest. This study establishes the ability of continuous low-intensity ultrasound (cLIUS) to preserve MSC chondrogenesis in a proinflammatory environment. MSCs were seeded in alginate:collagen hydrogels and cultured for 21 days in an ultrasound-assisted bioreactor (5.0 MHz, 2.5 Vpp; 4 applications/day) in the presence of IL1β and evaluated by qRT-PCR and immunofluorescence. The differential expression of markers associated with the NFκB pathway was assessed upon a single exposure of cLIUS and assayed by Western blotting, qRT-PCR, and immunofluorescence. Mitochondrial potential was evaluated by tetramethylrhodamine methyl ester (TMRM) assay. The chondroinductive potential of cLIUS was noted by the increased expression of SOX9 and COLII. cLIUS extended its chondroprotective effects by stabilizing the NFκB complex in the cytoplasm via engaging the IκBα feedback mechanism, thus preventing its nuclear translocation. cLIUS acted as a mitochondrial protective agent by restoring the mitochondrial potential and the mitochondrial mRNA expression in a proinflammatory environment. Altogether, our results demonstrated the potential of cLIUS for cartilage repair and regeneration under proinflammatory conditions.

## 1. Introduction

Damaged cartilage seldom heals; hence, therapies addressing restoration are of clinical relevance [1,2]. Strategies that rely on mesenchymal stromal cells (MSCs) to regenerate cartilage include mesenchymal-stromal-cell-implantation or microfracture, and depend upon the in-situ differentiation of MSC [3,4,5]. However, the inflamed environment caused by the surgical procedure itself, or by the diseased joint, [6] exerts a suppressive action on cartilage biosynthesis and the differentiation of MSCs into chondrocytes [7], and may explain inferior cartilage repair outcomes. Therapeutic measures that suppress the catabolic response in an inflamed environment by inhibiting key signaling mediators, including the nuclear factor kappa B (NFκB) pathway, are required to enable effective repair processes. Catabolic cytokines, interleukin-1beta (IL1β), tumor necrosis factor-alpha (TNFα), interleukin-6 (IL6), and interferon-gamma (IFNγ) were significantly elevated both in the synovial fluid and in the cartilage of diseased and operated joints [8]. IL1β- and TNFα-activated NFκB, and its downstream targets (MMP13, ADAMTS4/5), inhibited the biosynthesis of the cartilage matrix [9]. Activated NFκB also downregulated the expression of SOX9, the master regulator of chondrogenesis [10]. Not surprisingly, MSC chondrogenesis in pellet and 3D cultures were adversely impacted by the use of cytokines [11,12,13,14] and the gene delivery of IL1RA; culture additives like magnesium, melatonin, and curcumin were shown to rescue impaired MSC chondrogenesis [15,16,17,18].

Low-intensity ultrasound has been reported to enhance the chondrogenic differentiation of MSCS in vitro [19,20,21,22,23,24], albeit in the absence of cytokines. Specifically, our published work has shown that low-intensity continuous ultrasound (cLIUS), when employed at 14 kPa (5 MHz and 2.5 Vpp), was shown to positively regulate MSC chondrogenesis in vitro [25,26] by engaging SOX9 signaling pathways. Furthermore, the chondroprotective effects of cLIUS (14 kPa) against proinflammatory cytokines on adult chondrocytes was demonstrated, where cytokine-induced increases in NFκB expression and its downstream targets were suppressed, and the expression of collagen II (COLII) and TIMP1 genes were upregulated [27]. 

Since most early in vivo cartilage reparative processes occur in an inflammatory environment, this work focuses on evaluating the ability of cLIUS to mitigate the impairment of MSC chondrogenesis in a proinflammatory environment. Therefore, the assessment of MSC chondrogenesis in 3D hydrogels under cLIUS, in the presence of IL1β, was undertaken, where MSCs seeded in alginate:collagen hydrogels were cultured in the ultrasound-assisted bioreactor for 21 days and evaluated by quantitative real-time polymerase chain reaction (qRT-PCR), and immunofluorescence (IF). To quantify the NFκB pathway markers under cytokines and their differential expression under cLIUS, MSCs were subjected to a single exposure of cLIUS, and outcomes were evaluated by qRT-PCR, IF and Western blotting (WB). The ability of cLIUS to reverse the cytokine-induced impairment of mitochondrial potential (∆Ψm) was measured by tetramethylrhodamine methyl ester (TMRM) assay. 

## 2. Materials and Methods

### 2.1. Cell Culture

**Figure 1:**
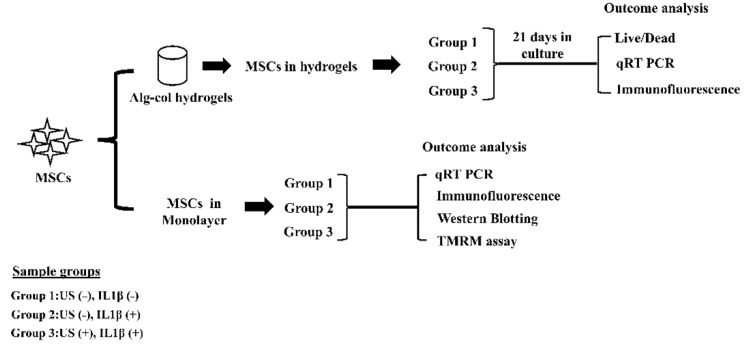
Experimental schematic. MSCs were seeded in alginate: collagen hydrogels or TCPs or coverslips, and divided into groups, as indicated. Group 1: cLIUS (−), IL1β (−); Group 2: IL1β (+), cLIUS (−); and Group 3: IL1β (+) cLIUS (+). Appropriate sample groups were treated with cytokine at a concentration of 10 ng/mL. cLIUS stimulation was applied as follows:14 kPa (5.0 MHz, 2.5 Vpp), and 10 or 20 min/application. Non-cytokine treated and non-cLIUS-stimulated samples served as controls. Upon completion of the study, samples were retrieved and subjected to the indicated outcome analyses.

Bone marrow-derived human MSCs (male, age 38) were purchased from Lonza (Walkersville, MD, USA) and expanded as previously reported [25]. Passage 3 to 5 MSCs were trypsinized and employed in all experiments. All cell culture experiments were carried out in humidified incubators at 37 °C with 5% CO_2_. The study design adopted is schematically shown in Figure 1.

### 2.2. Preparation and Encapsulation of MSCs in 3D Hydrogels

MSCs were encapsulated in alginate:collagen hydrogels at a seeding density of 2 × 10^5^ cells per hydrogel, and cultured for 21 days with and without IL1β (R&D systems, Minneapolis, MN, USA, 201-IL) or cLIUS stimulation. Briefly, a 0.4% sterile collagen solution (Advanced BioMatrix, Carlsbad, CA, USA, 5153) was mixed with neutralizing solution (Advanced BioMatrix, 5155) as per the manufacturer’s instructions. MSCs were added to the neutralized collagen solution at a ratio of 5:1, and mixed with 2% sterile alginate (Sigma, St. Louis, MO, USA, W201502) to yield a final concentration of 1.2% (alginate) and 0.11% (collagen). A previously reported protocol was adapted to prepare cell-laden hydrogels (5 mm × 5 mm), where 100 µL of the alginate-collagen-MSCs solution was pipetted into 5 mm × 5 mm agarose molds containing 0.5 M CaCl_2_, and incubated at 37 °C for 30 min [28]. Formed hydrogels were removed and cultured in six-well tissue culture plates (TCPs) in α-MEM basal media supplemented with 10% fetal bovine serum, 1 × Glutamax^TM^ and 1 × Antibiotic- Antimyotic^TM^ (Gibco, Waltham, MT, USA) solution for 72 h.

### 2.3. MSCs Culture in Hydrogels and cLIUS Treatment

Hydrogels were divided into three sample groups as follows: Group 1: cLIUS (−), IL1β (−); Group 2: IL1β (+), cLIUS (−); and Group 3: IL1β (+) cLIUS (+) and schematically depicted in Figure 1. Media was replaced every 3 days, where only half of the media was supplemented with fresh media containing 10 ng/mL of IL1β. Automated cLIUS stimulation was applied using the bioreactor [23,29,30] developed and characterized at the University of Alabama in Huntsville (UAH) at the following regimen: 14 kPa (5.0 MHz, 2.5 Vpp), 10 min/application, and four applications/day. Hydrogels were retrieved at the end of 21 days and subjected to outcome analyses as listed in Figure 1.

### 2.4. MSCs Culture in Monolayer and cLIUS Treatment 

MSCs were plated in 6-well or 12-well TCP at the following seeding densities: 2 × 10^5^ or 5 × 10^4^ cells/well (protein and RNA extractions following treatment with cytokines and non-treated controls) and 1 × 10^4^ cells/coverslip (CS) (for TMRM assay and IF studies following treatment with cytokines and non-treated controls). All treatments with cytokines and/or cLIUS were conducted after 48 h of initial seeding of MSCs in TCP or CS. All cytokines were purchased from R&D systems (Minneapolis, MN, USA) and were employed at a concentration of 10 ng/mL. Non-focused immersion transducers (Panametrics V300, 12.7 mm diameters, Panametrix, Waltham, MA, USA) were used to apply cLIUS to plated MSCs using procedure detailed elsewhere [23,29,30]. MSCs were exposed to cytokines and cLIUS was applied one time for 10 or 20 min at 5 MHz (2.5 Vpp) with a constant pressure amplitude of 14 kPa. In addition to qRT-PCR following cLIUS stimulation, WB, TMRM assay and IF staining were conducted.

### 2.5. Cell Viability Assay

Cell viability in hydrogels was assessed by Live/Dead^TM^ Viability/Cytotoxicity kit (Molecular Probes, Eugene, OR USA) according to manufacturer’s instructions [27] and visualized with the Zeiss LSM 700 confocal microscope. All the images were collected at 10× magnification (z step size = 12 μm).

### 2.6. Quantitative Real-Time PCR (qRT-PCR)

MSCs were released from hydrogels (*n* = 10 hydrogels per sample) using the dissolution buffer (DB) (55 mM sodium citrate, 50 mM EDTA, and 90 mM NaCl, pH 6.9) and homogenized with the TRIzol reagent (Invitrogen, Waltham, MT, USA). In monolayer studies, cells from TCP plates were homogenized with 300 µL of TRIzol reagent per well. Homogenates from two wells served as one replicate, and three such replicates were used for gene expression analysis (*n* = 3). RNA was extracted using PureLink RNA Mini Kit (Thermofisher, Waltham, MT, USA). The qRT-PCR analysis was carried out using QuantStudio 3 real-time PCR system (Applied Biosystems, Waltham, MT, USA) employing TaqMan^®^ RNA-to-CT™ 1-Step Kit (Life Technologies, Waltham, MT, USA). 

TaqMan^®^ Gene expression assays (Life Technologies, USA) used are as follows:

GAPDH (Hs02786624_g1), MMP13 (Hs00942584_m1), ADAMTS4 (Hs00192708_m1), NFκB (Hs00765730_m1), TIMP1 (Hs01092512_g1), SOX9 (Hs00165814_m1), RUNX2 (Hs01047973_m1), PPARG (Hs01115513_m1) and MTCO3 (Hs02596866_g1), MTCYB (Hs02596867_s1). The expression of mRNA transcripts was normalized to GAPDH expression and relative expression levels were calculated using the 2^–ΔΔCt^ method.

### 2.7. Immunofluorescence Staining

MSCs on CS were fixed in 4% paraformaldehyde (4% PFA) for 20 min and blocked with 2% goat serum in 1X TBST (Tris-buffered saline with 0.1% tween20) blocking buffer (BB) for 2 h. CS were then incubated with 1:1000th diluted rabbit anti-phospho-NFκB p65 monoclonal antibody (Invitrogen, MA5-15160) in BB overnight at 4 °C. Upon washing, CS were incubated with 1:1000th diluted goat anti-rabbit IgG H&L conjugated with Alexa flour 488 (Abcam, 150077) for 1 h at room temperature (RT) and mounted on a glass slide with DAPI mounting media (ProLong™ Diamond Antifade Mountant with DAPI, P36962). For CS subjected to double IF staining, CS treated as above till the NFκB detection step and then washed and blocked with BB for 1 h at RT and incubated with 1:1000th diluted rabbit anti-human SOX9 Mab (CST, 82630) overnight at 4 °C. Upon washing, CS were incubated with 1:1000th diluted goat anti-rabbit IgG H&L conjugated with Alexa flour 594 (CST, 8889S), for 1 h at RT and mounted as mentioned earlier. All images were captured using the Zeiss LSM 700 confocal microscope at 63-times magnification. Fluorescent intensities were quantified using ImageJ^TM^ software (*n* = 30–60).

To visualize pNFκB and COLII in hydrogels, a modified IF staining protocol was adopted [31], where hydrogels were washed in HBSSCM (HBSS containing 1.26 mM CaCl_2_ and 0.4 mM MgSO_4_) and fixed with 4% PFA containing 1.26 mM CaCl_2_, 400 mM MgSO_4_ for 60 min. After copious washing, samples were permeabilized with 0.1% Triton X-100 in HBSSCM and blocked with BB (5% BSA, 10% goat serum in HBSSCM containing 0.2% tween) for 2 h at RT. pNFκB and COLII were independently stained and detected as mentioned earlier. COLII was detected using 1:1000th diluted rabbit anti-human collagen II polyclonal antibody (Abcam, 34712) and Alexa flour 488 conjugated goat anti-rabbit polyclonal antibody. All images were collected at 63-times magnification using the Zeiss LSM 700 confocal microscope (z stacks: 180–190 µm and z step size: 5 µm), and fluorescent intensity was quantified using ImageJ^TM^ software (*n* = 25).

### 2.8. Protein Isolation and Western Blotting

Total protein was extracted and quantified upon cessation of cLIUS stimulation, using previously published methods [24]. Lysates from three independent wells were pooled together for total protein extraction (*n* = 6). SDS-PAGE was conducted using Novex^TM^ Tris-Glycine gels (Invitrogen, USA) per the manufacturer’s instructions. Proteins separated by SDS-PAGE were transferred to the PVDF membrane. Membranes were blocked with 5% dry milk (CST, 9999) for 1 h at RT and incubated with 1:1000th diluted primary antibodies of pNFκB (CST, 3033), tNFκB (CST, 8242), pIκBα (CST, 2859), and tIκBα (CST, 4814) in 5% BSA overnight at 4 °C. Detection was performed by incubating with 1:2000th diluted horse-radish-peroxidase (HRP) labeled secondary antibodies to rabbit IgG (CST, 7074) and mouse IgG (CST, 7076). β-actin was used as a loading control. The cytoplasmic and nuclear fractions were isolated using NE-PER™ kit (Thermo Scientific, Waltham, MT, USA) and processed for Western blotting as described. All blots were visualized by incubating with Clarity™ Western ECL kit (Bio-Rad, Hercules, CA, USA) as per the manufacturer’s instructions. Images were captured with a ChemiDoc MP imaging system (Bio-Rad, Hercules, CA, USA), and the band intensities were quantified using ImageJ™ software.

### 2.9. Measurement of Mitochondrial Potential Using TMRM Assay

Mitochondrial potential (ΔΨm) in various study groups (Figure 1) was assessed using the TMRM assay as per manufacturer’s instructions. Briefly, cells on CS were washed and incubated with 100 nM TMRM reagent for 30 min. Live images were captured using Zeiss LSM 700 confocal microscope at 10× magnification, and fluorescent intensity was quantified using ImageJ^TM^ software (*n* = 50). 

### 2.10. Statistical Analysis

The data are expressed as average ± standard deviation. All experimental data was analyzed using one-way ANOVA followed by post-hoc Sidak’s multiple comparison test. The graphs were generated using GraphPad Prism software. Statistical significance was established as follows: *p* < 0.05 (denoted as *), *p* < 0.01 (denoted as **), *p* < 0.001 (denoted as ***), *p* < 0.0001 (denoted as ****).

## 3. Results

The chondroinductive potential of cLIUS was reported elsewhere, albeit in the absence of cytokines [24,25,26]. Thus, to examine the chondroinductive ability of cLIUS in a cytokine rich environment, chondrogenic differentiation of MSCs was evaluated in alginate:collagen hydrogels (as shown in Figure 1). Live dead assay was used to assess the cellular viability in hydrogels on day 21 and this is shown in Supplementary Figure S1. Good cell viability was observed in all the study groups, and no appreciable levels of dead cells (red) were observed. 

### 3.1. cLIUS Preserves Collagen II Protein Expression in the Presence of Cytokines

**Figure 2:**
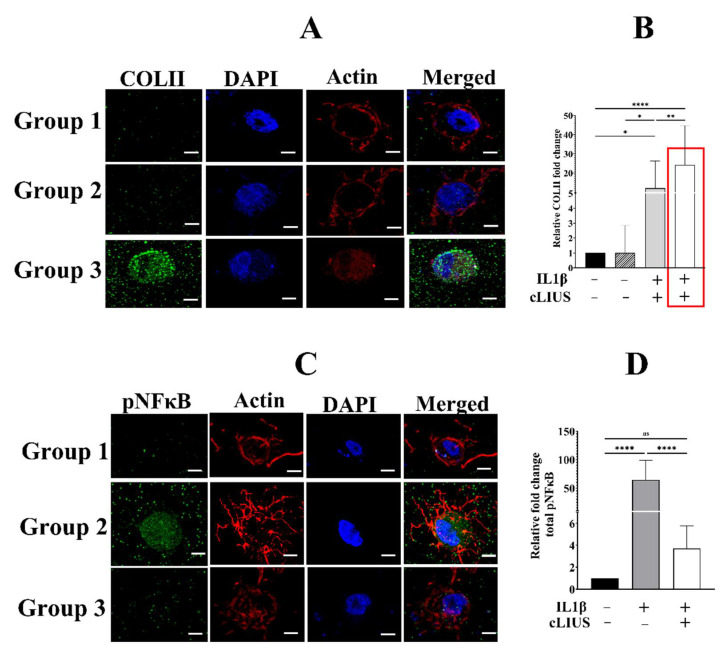
Localization of pNFκB and COLII in hydrogel scaffolds. At the end of 21 days of culture, hydrogels were subjected to both gene expression and IF analysis. Briefly, hydrogels were fixed using 4% PFA in HBSSCM and stained against COLII and pNFκB (green fluorescence), respectively, in separate experiments (**A**,**C**), and nuclei were counter stained with DAPI (blue fluorescence). Z stacks of the hydrogels were captured using the Zeiss LSM 700 confocal microscope with 63× magnification (z step size 5 µm), and fluorescence intensity (**B**,**D**) was quantified using ImageJ^TM^ software (*n* = 30). Data are shown as the mean ± standard deviation of samples and *p*-value represents statistical significance (* *p* < 0.05; ** *p* < 0.01; **** *p* < 0.0001 and ns-nonsignificant) and scale bar represents 5 µm. ‘+’ indicates presence and ‘–’ indicates absence.

Similar to previously reported studies, a 24-fold higher protein expression level of COLII was noted in samples treated with cLIUS alone, compared to controls (boxed red line in Figure 2B). The representative images are presented in Supplementary Figure S2B. Catabolic cytokines are known to inhibit extracellular matrix synthesis, especially COLII [9]. Hence, the ability of cLIUS to maintain COLII expression in a IL1β rich environment was ascertained upon culture in alginate:collagen hydrogels. A 12-fold increase in COLII expression was observed in group 3 samples compared to group 1, thus indicating the ability of cLIUS to support MSC chondrogenesis in the presence of cytokines. 

### 3.2. cLIUS Abrogates pNFκB Nuclear Translocation Induced by IL1β in 3D Scaffolds

For activated NFκB to induce deleterious catabolic effects, translocation of pNFκB to the nucleus is necessary [32]. As cLIUS was noted to mitigate the cytokine (IL1β)-mediated catabolic effects, experiments were undertaken to visualize the localization and nuclear translocation of phosphorylated NFκB (pNFκB) in hydrogels via IF, and these are presented in Figure 2C. Compared to the control (group 1), a 38-fold higher level of pNFκB intensity in the cytoplasm was noted in IL1β treated samples (group 2). The application of cLIUS, in the presence of IL1β (group 3), decreased the cytoplasmic pNFκB intensity to levels observed in group 1. The inclusion of IL1β led to enhanced localization of pNFκB in the nucleus when compared to controls. cLIUS stimulation (group 3) diminished the intensity of pNFκB in the nucleus to levels observed in controls, indicating that cLIUS blunted the translocation of pNFκB to the nucleus in the presence of IL1β. cLIUS had no discernible impact on the expression of NFκB pathway markers (Supplementary Figure S2A).

### 3.3. cLIUS Attenuates NFκB Expression and Upregulates SOX9 Gene Expression in the Presence of IL1β

**Figure 3:**
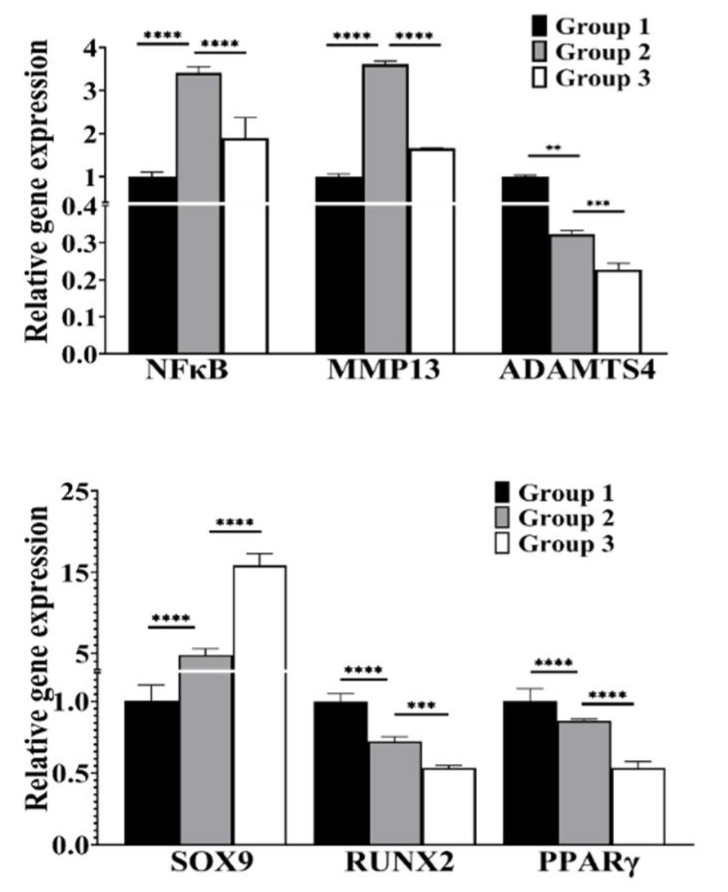
Gene expression analysis of MSCs in hydrogels. Cell homogenates (*n* = 10) were prepared from hydrogels; total RNA was extracted, and gene expression of lineage markers and catabolic markers was evaluated by qRT-PCR, and GAPDH was used as a housekeeping gene. Data are shown as the mean ± standard deviation of samples and *p*-value represents statistical significance (** *p* < 0.01; *** *p* < 0.001 and **** *p* < 0.0001).

Catabolic cytokines activate NFκB, leading to the downregulation of SOX9, the main collagen transcription factor [10]. To ascertain the ability of cLIUS to support the chondrogenic differentiation of MSCs in the presence of cytokines, the gene expression of catabolic markers (NFκB, MMP13, ADAMTS4) and lineage markers (RUNX2, PPARγ, and SOX9) was evaluated by qRT-PCR, and this is shown in Figure 3. As expected, the presence of IL1β (group 2) significantly elevated the gene expression of MMP13 (3.6 fold) and NFκB (3.4 fold) when compared to controls (group 1). cLIUS stimulation (group 3) significantly diminished IL1β-induced upregulation of these markers. A 15-fold higher expression of SOX9 was noted in group 3, where IL1β-treated samples were exposed to cLIUS when compared to IL1β-treated samples (group 2). Interestingly, low gene expression levels of osteogenic and adipogenic differentiation markers (RUNX2 and PPARγ) were noted in cLIUS-stimulated samples with IL1β treatment. 

Since molecular consequences following cLIUS on the canonical NFκB pathway markers are best evaluated upon a single exposure of cells to cLIUS in monolayer experiments, MSCs in monolayers were subjected to the study design shown in Figure 1.

### 3.4. cLIUS Downregulates Catabolic and Upregulates Anabolic Gene Expression in the Presence of Proinflammatory Cytokines

**Figure 4:**
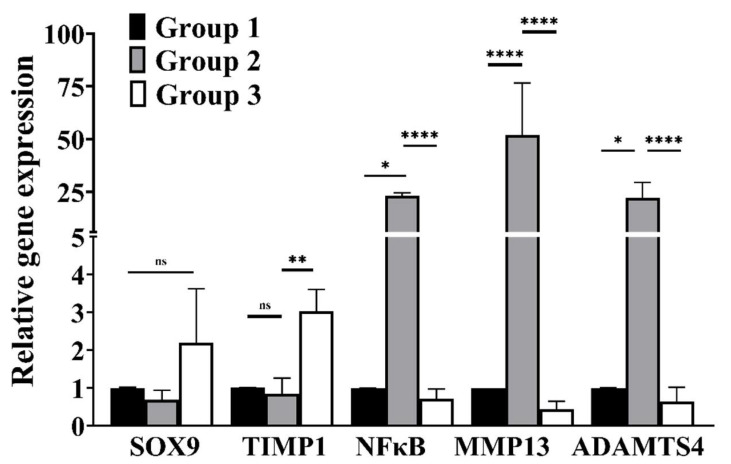
Gene expression analysis in MSCs exposed to IL1β. MSCs were seeded on TCPs and treated as depicted in Figure 1. Homogenates from two wells per group served as one replicate, and three such replicates were used for gene expression analysis (*n* = 3). Total RNA was extracted, and the gene expression of anabolic and catabolic markers was evaluated by qRT-PCR; GAPDH was used as a housekeeping gene. Bar graph represents mean ± 95% confidence interval; *p* values indicate statistically significant differences (* *p* < 0.05; ** *p* < 0.01; **** *p* < 0.0001 and ns-nonsignificant).

The gene expression levels of catabolic (MMP13, ADAMTS4), anabolic (TIMP1), and transcription markers (NFκB and SOX9) were evaluated by qRT-PCR and the results shown in Figure 4. In the presence of IL1β (group 2), the expression of catabolic genes and NFκB was significantly elevated, and the gene expression of SOX9 and TIMP1 were downregulated compared to controls (group 1). When IL1β-treated cells were further exposed to cLIUS, an abrogation in the expression levels of catabolic genes to basal levels, similar to group 1, was noted. However, in the same sample treatment (group 3), cLIUS yielded high gene expression levels of SOX9 and TIMP1. Independent experiments were also carried out in the presence of TNFα or IL6, and similar trends were noted and presented in Supplementary Figure S3. Cumulative results demonstrated that cLIUS promotes the gene expression of anabolic markers in the presence of proinflammatory cytokines by downregulating catabolic genes MMP13, ADAMTS4, and NFκB.

### 3.5. cLIUS Rescinds pNFκB and Promotes SOX9 Localization to the Nucleus in the Presence of IL1β

**Figure 5:**
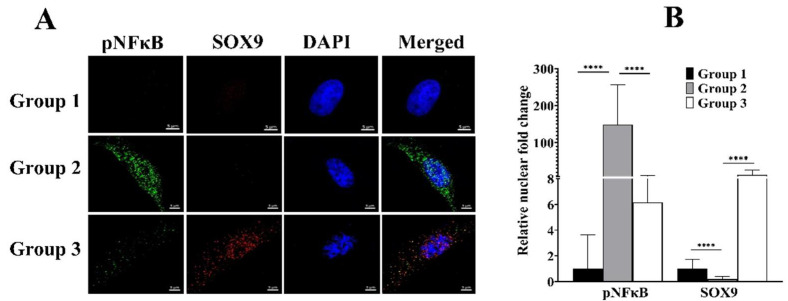
Localization of SOX9 and pNFκB in MSCs exposed to IL1β. MSCs were seeded on coverslips and treated as depicted in Figure 1. Coverslips were fixed and double stained for pNFκB (green fluorescence) and SOX9 (red fluorescence) antibodies, and nuclei were counter stained with DAPI (blue fluorescence). (**A**) Images were captured using 63× magnification and presented. (**B**) Fluorescence intensity was quantified using ImageJ^TM^ software (*n* = 30). Bar graph represents mean ±95% confidence interval; *p* values indicate statistically significant differences. (**** *p* < 0.0001) and the scale bar represents 5 µm.

To evaluate the influence of cLIUS on IL1β-induced cellular localization of pNFκB, IF studies were undertaken and presented in Supplementary Figure S4A. As expected, the presence of IL1β (group 2) yielded a 21-fold higher intensity of pNFκB in the cytoplasm and nuclear region when compared to controls (group 1). The cytoplasmic and nuclear intensity levels of pNFκB were significantly diminished when cells were exposed to cLIUS stimulation in the presence of IL1β (group 3). Comparable trends were noted when experiments were undertaken in the presence of TNFα or IL6 (Supplementary Figure S4B,C). Since the nuclear localization of pNFκB is linked to its proinflammatory transcriptional activity, localization of pNFκB and SOX9 in the cytoplasmic and nuclear regions were visualized by double IF, and the fluorescent intensities were quantified and presented in Figure 5. The presence of IL1β reduced the levels of SOX9 in the nucleus (group 2). However, cLIUS stimulation in the presence of IL1β (group 3) inverted the localization of these markers, where the levels of nuclear pNFκB were decreased, and SOX9 was upregulated. Similar trends were observed in cytoplasmic levels of pNFκB and SOX9. These results indicate the ability of cLIUS to maintain the expression of SOX9, the main transcription factor of COL2A1, in the presence of proinflammatory cytokines.

### 3.6. cLIUS Minimizes IL1β Induced pNFκB Expression and Persuades Total IκBα Expression

**Figure 6:**
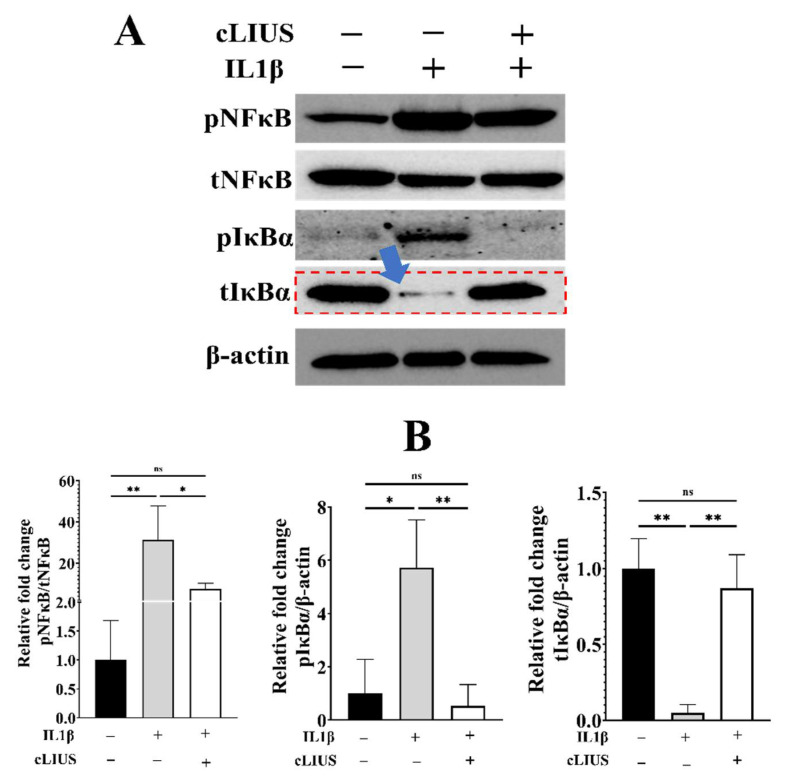
Phosphorylation of NFκB and IκBα. MSCs were seeded on TCP plates and treated as depicted in Figure 1. Total cell lysates were obtained and analyzed by Western blotting using specific antibodies. (**A**) Protein expression of phospho-NFκB, total NFκB, phospho-IκBα and total IκBα in indicated samples by Western blotting; β-actin was used as a loading control. (**B**) Blots were quantified using ImageJ^TM^ software and presented. Data are shown as the mean ± standard deviation of samples and *p*-value represents statistical significance (* *p* < 0.05; ** *p* < 0.01; and ns-nonsignificant). ‘+’ indicates presence and ‘–’ indicates absence.

To ascertain the ability of cLIUS to deactivate the NFκB pathway in MSCs exposed to IL1β, protein expression of NFκB pathway markers (phosphorylated NFκB (pNFκB), total NFκB (tNFκB), phosphorylated IκBα (pIκBα), and total IκBα (tIκBα)) was analyzed by Western blotting and shown in Figure 6. In IL1β, treated samples (group 2), a 31-fold higher level of pNFκB expression and a 6-fold elevated level of pIκBα expression was observed in comparison to non-treated controls (group 1). The expression level of tIκBα was significantly downregulated in the presence of IL1β (indicated by the blue arrow in Figure 5A). However, cLIUS stimulation (group 3) diminished the IL1β upregulated expression of these transcription factors to control levels. Notably, when IL1β-treated cells were exposed to cLIUS (group 3), the expression level of tIκBα was similar to that observed in controls (indicated by the red dotted lines in Figure 5A). The significant increase in tIκBα expression levels indicates cLIUS-induced suppression of the NFκB pathway in the presence of IL1β by engaging the tIκBα feedback mechanism, thus rescuing MSCs from the negative impact of activated NFκB. cLIUS alone does not activate the NFκB pathway as noted by the similar expression levels of NFκB pathway markers between control and cLIUS samples (Figure S5). 

### 3.7. cLIUS Acts as a Mitochondrial Protective Agent in the Presence of IL1β

**Figure 7:**
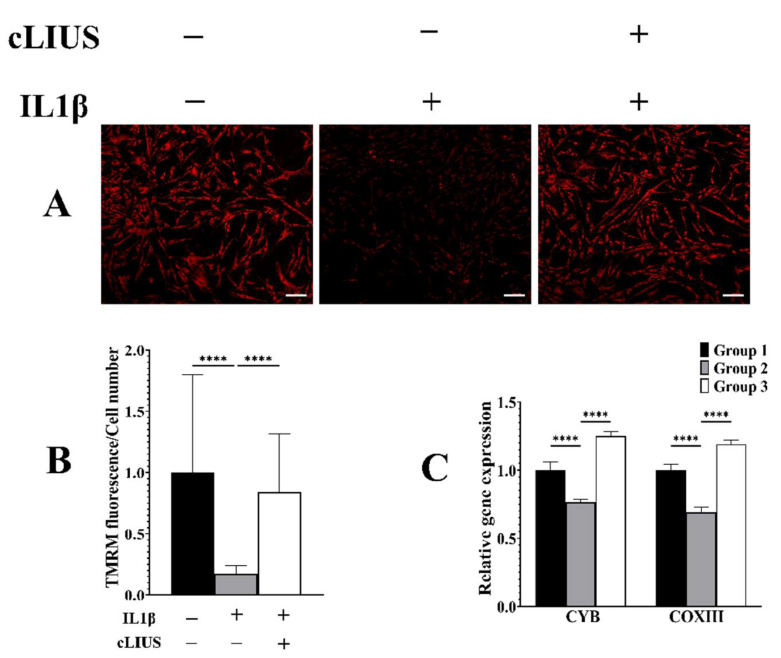
Assessment of mitochondrial potential and mRNA expression under cLIUS. MSCs were seeded on coverslips and treated as depicted in Figure 1. Coverslips were treated with 100 nM TMRM reagent for 30 min, and live images were captured using the Zeiss LSM 700 confocal microscope, and images are presented in (**A**). Fluorescence data was quantified (*n* = 50) using ImageJ^TM^ software and the fluorescence intensity graph presented in (**B**) along with mitochondrial gene expression (**C**). All data shown as the mean ± standard deviation of samples and *p*-value represent statistical significance (**** *p* < 0.0001), and scale bar represents 100 µm. ‘+’ indicates presence and ‘–’ indicates absence.

There is increasing evidence for the presence of NFκB in the mitochondria of cells that are exposed to cytokines, and this has been shown to lower the ΔΨm [33]. TMRM assay was employed to ascertain the ΔΨm under cLIUS and in the presence of IL1β, and both the fluorescent images and their quantification are shown in Figure 7. cLIUS alone had no discernable impact on ΔΨm when compared to controls (data not included). This is supported by our previous observation that cLIUS regimens employed in this study do not generate reactive oxygen species [23]. As expected, the ΔΨm was significantly downregulated in IL1β-treated samples (group 2) when compared to controls (group 1). The IL1β-induced decrease in ΔΨm was reinstated by cLIUS stimulation (group 3) as demonstrated by a significant (*p* < 0.0001) increase in ΔΨm (Figure 6B). Previously, the presence of NFκB in mitochondria was noted to lower the expression of Cytochrome c oxidase Ⅲ (COXIII) and Cytochrome b (CYB) [33]. Thus, gene expression of COX Ⅲ and CYB mRNA levels were used as an indirect measure of the NFκB in the mitochondria, and evaluated by qRT-PCR, and this is presented in Figure 7C. In the presence of IL1β (group 2), both COX Ⅲ and CYB levels were decreased when compared to controls (group 1). When IL1β-treated cells were exposed to cLIUS (group 3), the gene expression levels of COXIII and CYB were unregulated, as noted in controls.

## 4. Discussion

It is well-established that IL1β and TNFα are upregulated in diseased and operated joints and exert catabolic effects via the canonical and non-canonical NFκB pathway0s [11]. Cytokine-induced activation of NFκB downregulated the key chondrogenic transcription factor, SOX9, and upregulated the expression of matrix-degrading proteins (MMP13, ADAMTS4), [34,35] thus impeding chondrogenesis. Therefore, the resulting imbalance of the anabolic processes in a proinflammatory environment inhibits MSC chondrogenesis, leading to inferior cartilage repair outcomes. Hence, strategies to rescue MSC chondrogenesis under proinflammatory conditions are of interest. In that regard, select natural compounds with known anti-inflammatory properties were shown to rescue MSC chondrogenesis in the presence of IL1β by suppressing the activation of NFκB and maintaining the expression of chondrocyte markers [15,18]. Treatment of MSC pellets or MSC cell aggregates with melatonin or IL1RA expression resulted in elevated expression of GAG synthesis and matrix proteoglycans, which were negatively impacted by the cytokines [15,17]. Similarly, delivery of siRNA, the addition of divalent ions, and hypoxic culture conditions helped rescue chondrogenesis of MSCs in a proinflammatory environment by suppressing the activation of NFκB [11,16,36].

Similarly to previously reported studies, elevated levels of COLII expression were noted in MSCs exposed to cLIUS, reaffirming the chondroinductive potential [24,25]. To the best knowledge, this is the first report that demonstrates the ability of cLIUS to abrogate the deleterious impact of IL1β and preserve MSC chondrogenesis by maintaining elevated levels of COLII while downregulating the activity of NFκB. In the present study, increased nuclear deposition of SOX9 and its elevated gene expression in the presence of IL1β, indicates the chondroinductive ability of cLIUS. cLIUS further extended its chondroinductive potential by down regulating the cytokine (IL1β, TNFα and IL6)-induced catabolic responses (MMP13, ADAMTS4 and NFκB) and upregulating anabolic responses (SOX9, TIMP1), irrespective of cytokines tested in this study.

Under inflammatory conditions, NFκB is the main transcription factor that induces the expression of catabolic genes [34,35]; strategies that aim to rescue MSC chondrogenesis are typically focused on the canonical pathways of NFκB activation. In normal cells, the NFκB complex exists in the cytoplasm as an NFκB dimer bound to its inhibitor protein tIκB, which impedes NFκB DNA-binding activity and prevents its nuclear translocation. In the presence of inflammatory stimuli, signal-dependent phosphorylation of NFκB and IκB proteins led to the dissociation of the NFκB complex and the subsequent nuclear translocation of the NFκB dimer that induced the transcription of inflammatory genes. Phosphorylated IκBα is then designated for ubiquitination and degradation [37]. In the present study, as anticipated, the inclusion of IL1β in the culture led to increased levels of pNFκB and pIκBα, indicating increased activity of IKK [37] with a concomitant reduction of tIκBα levels. cLIUS blunted the expression of pNFκB and pIκBα, indicating reduced IKK activity. Surprisingly cLIUS restored the levels of tIκBα. Low levels of pIκBα and high levels of tIκBα in group 3 samples indicated that the NFκB complex is stabilized under cLIUS by hindering the IκBα phosphorylation; similar trends were observed by Cogswell et al., [33]. This study documents the ability of cLIUS to preserve MSC chondrogenesis by downregulating NFκB activity via the IκBα-assisted negative feedback regulation.

Chondrocytes, when exposed to proinflammatory cytokines (i.e., IL1β, TNFα) in vitro (as in an osteoarthritic joint environment), showed a reduction in enzyme mitochondrial activities of complexes II and III, as well as a reduction in ΔΨm [33]. In particular, the expression of mitochondrially encoded COX Ⅲ and CYB mRNAs was reduced by cytokines [33]. When the activation of mitochondrial NFκB was inhibited by the expression of the super-repressor form of IκBα, the expression of both COX III and CYB mRNA returned to normal levels [33]. These data indicate that the NFκB regulatory pathway exists in mitochondria and that NFκB levels can be negatively correlated to mitochondrial mRNA expression. Thus, mRNA levels of COX III and CYB were used as an indirect indication of the presence of NFκB in the mitochondrion when treated with cytokines [33]. As anticipated, our results showed that in IL1β treated samples, expression of both COX III and CYB mRNA were downregulated when compared to controls; furthermore, the ΔΨm was reduced, alluding to the presence of NFκB in the mitochondria. Interestingly, cLIUS acts as a mitochondrial protective agent, in that it restores both the ΔΨm and the mRNA levels of COX III and CYB). To our knowledge, mitochondrial protective ability of cLIUS has not been previously reported. It is possible that cLIUS blunts the translocation of pNFκB to the mitochondria, or it abolishes the activity of pNFκB in the mitochondria. These aspects will be investigated in greater detail in our ongoing investigations. 

## 5. Conclusions

**Figure 8:**
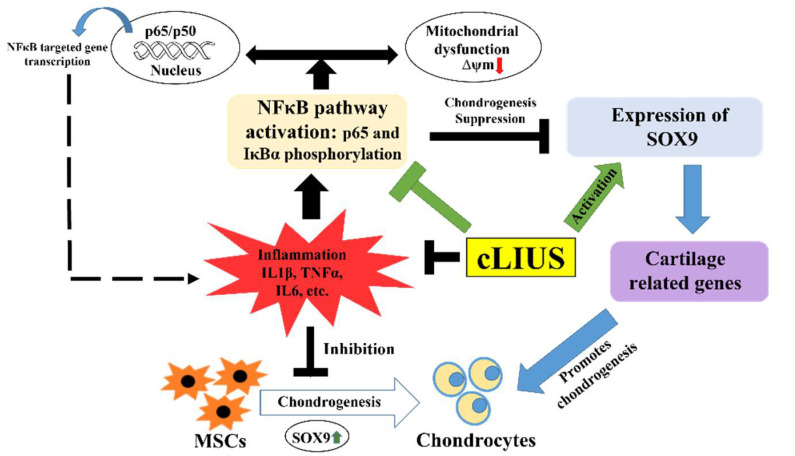
Schematic representation of the cLIUS-induced chondroprotective mechanisms. cLIUS promoted MSC chondrogenesis by inhibiting the cytokine-induced activation of the NFκB signaling pathway by engaging the tIκBα feedback mechanism while upregulating the expression of SOX9, the collagen II transcription factor. It is also posited that cLIUS acts to preserve the mitochondrial potential, which is impacted by cytokines.

In summary, this study reinforces the ability of cLIUS to preserve MSC chondrogenesis in a proinflammatory environment by taking advantage of the IκBα feedback mechanism to inhibit the nuclear translocation of NFκB, as well as protecting the mitochondrial potential and mitochondrial mRNA expression (Figure 8). Results will be further validated using MSCs from four to six different sources. Future studies will focus on the in-depth evaluation of mitochondrial dynamics under cLIUS in a proinflammatory environment, together with a long-term culture for 56 days that will allow an in-depth characterization of matrix synthesis and biochemical properties, similar to as previously reported [26]. This study establishes the potential of cLIUS to improve and enhance outcomes of in vivo cartilage repair therapies. Translation of promising in vitro findings with cLIUS requires an understanding of the cLIUS propagation in the joint space, together with optimal transducer settings. Current efforts are focused on establishing relevant mathematical models to allow for translation to small animal cartilage repair models to demonstrate the utility of cLIUS in improving cartilage repair outcomes.

## Data Availability

Not applicable.

## Supplementary Materials

The following are available online at https://www.mdpi.com/article/10.3390/biom12030434/s1, Figure S1: Cell viability of hydrogel scaffolds. Figure S2: Localization of pNFκB and COLII in cLIUS treated and non-treated control hydrogel scaffolds. Figure S3: Gene expression analysis in MSCs exposed TNFα (A) and IL6 (B). Figure S4: Localization of pNFκB in MSCs exposed to cytokines IL1β (A), TNFα (B) and IL6 (C). Figure S5: Phosphorylation of NFκB and IκBα in cLIUS treated and non-treated control samples.
