# Supplementary material for: Continuous Low-Intensity Ultrasound Preserves Chondrogenesis of Mesenchymal Stromal Cells in the Presence of Cytokines by Inhibiting NFκB Activation
Source: Biomolecules. 2022 Mar 11;12(3):434. doi: 10.3390/biom12030434 (PMC8946190; doi:10.3390/biom12030434)
Supplement: Supplementary file 1 [file biomolecules-12-00434-s001.zip › biomolecules-1561709-supplementary.pdf]

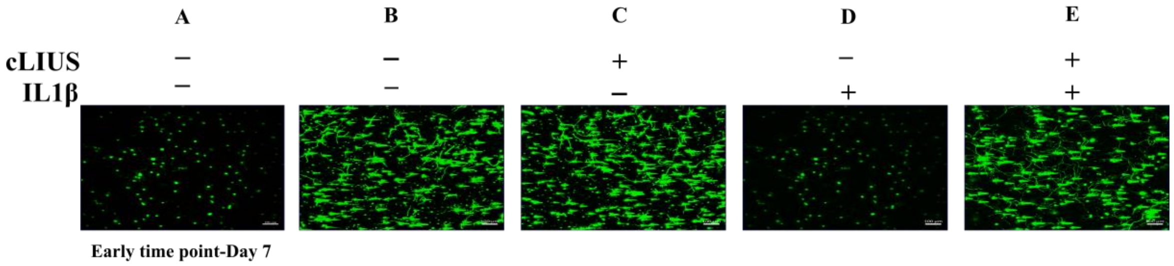

Figure S1. Cell viability of hydrogel scaffolds: Hydrogels were processed for Live/Dead analyses via Calein™ staining and imaged at 10X magnification (scale bar: 100 µm). Live cells stained green with Calcein^{AM} and dead cells stained red with ethidium homodimer-1. [A] A cell-laden hydrogel sample from an early time point. [**B** to **E**] Cell laden hydrogels from various groups as indicated.

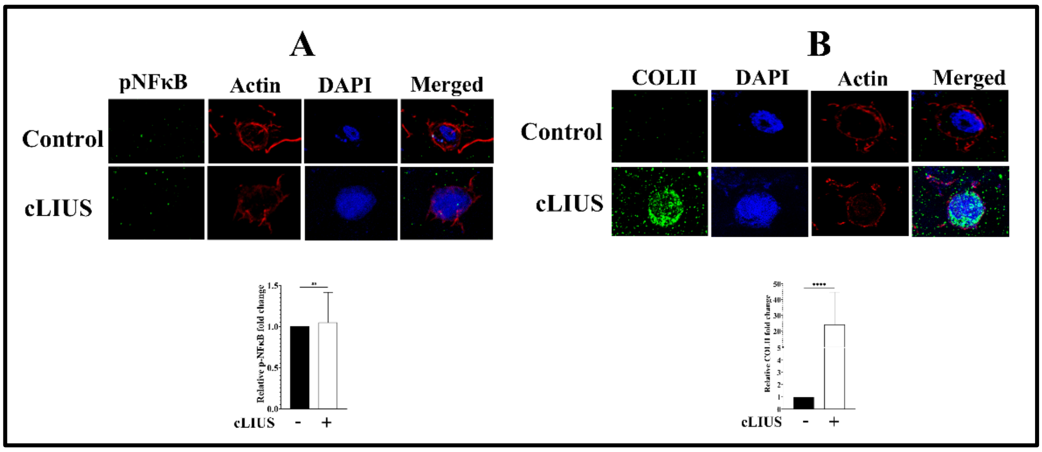

Figure S2. Localization of pNFκB and COLII in cLIUS treated and non treated control hydrogel scaffolds: At the end of 21days, , in separate experiments Hydrogels were fixed using 4% PFA in HBSSCM and stained against pNFκB and COLII (green fluorescence) (**A**, **B**) respectively, and nuclei were counter stained with DAPI (blue fluorescence). Z stacks of the hydrogels were captured using the Zeiss LSM 700 confocal microscope with 63x magnification (z step size 5µm), and fluorescence intensity was quantified using ImageJ™ software (n=30). Data are shown as the mean ± standard deviation of samples and p-value represents statistical significance (p*< 0.05; p**< 0.01; p***<0.001 and p****<0.0001).

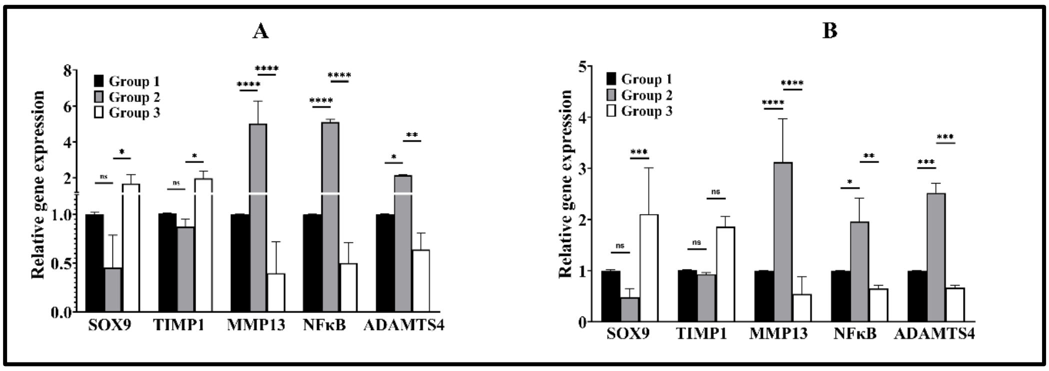

Figure S3. Gene expression analysis in MSCs exposed TNFα(A) and IL6(B). MSCs were seeded on TCPs and treated as depicted in Figure 1. Homogenates from two wells per group served as one replicate and three such replicates were used for gene expression analysis (n = 3). Total RNA was extracted, and the gene expression of anabolic and catabolic markers was evaluated by qRT-PCR and GAPDH was used as a housekeeping gene. Bar graph represents mean ± 95% confidence interval; p values indicate statistically significant differences (p*< 0.05; p**< 0.01; p***<0.001 and p****<0.0001).

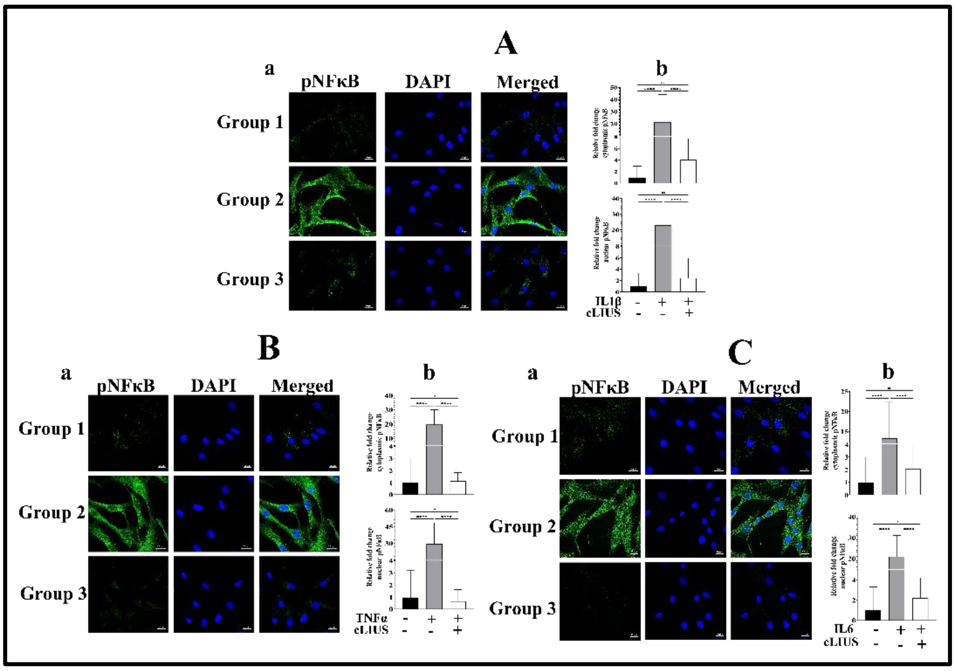

Figure S4. Localization of pNFκB in MSCs exposed to cytokines IL1β (A), TNFα (B) and IL6 (C). MSCs were seeded on coverslips and treated as depicted in Figure 1. Coverslips were fixed and stained for pNFκB (green fluorescence), and nuclei were counter stained with DAPI (blue fluorescence). (a) Images were captured using 63x magnification and presented. (b)Fluorescence intensity was quantified using ImageJ™ software (n=60). Bar graph represents mean ± 95% confidence interval; p values indicate statistically significant differences. (p*< 0.05; p**< 0.01; p***<0.001 and p****<0.0001) and the scale bar represents 20μm.

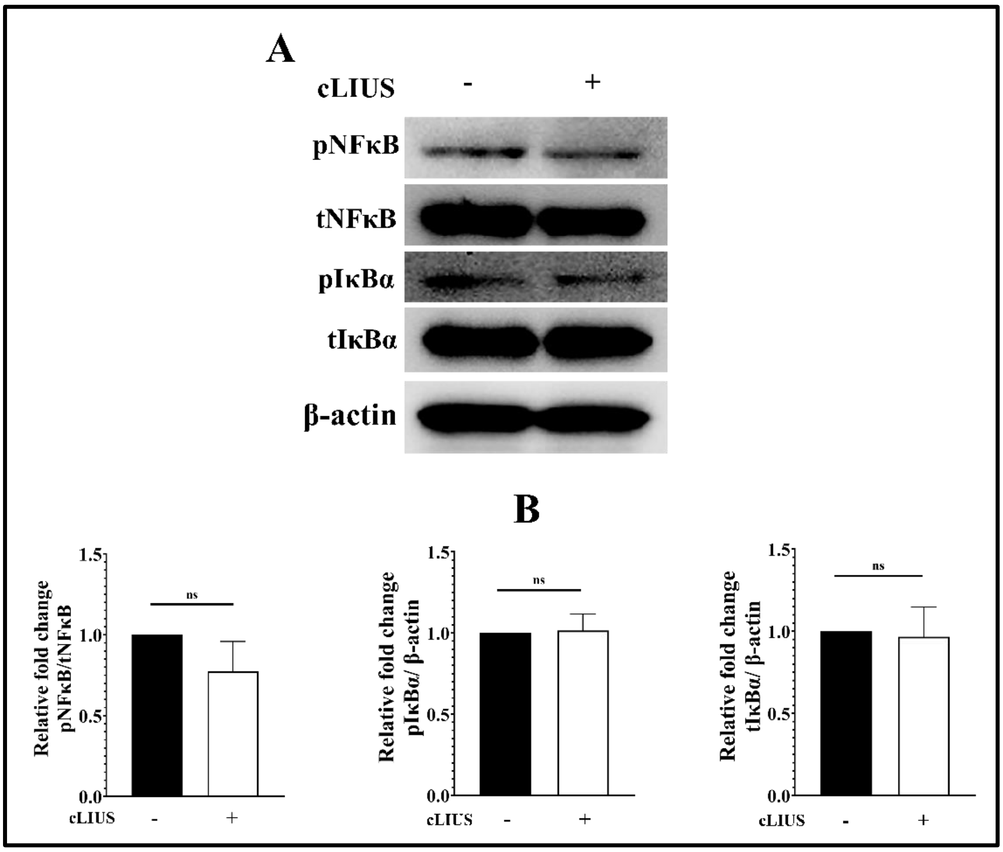

Figure S5. Phosphorylation of NFκB and IκBα in cLIUS treated and non treated control samples. MSCs were seeded on TCP plates and treated as depicted in Figure 1. Total cell lysates were obtained and analyzed by western blotting using specific antibodies. (**A**) Protein expression of phospho-NFκB,

total NFκB, phospho-IκBα and total IκBα in indicated samples by western blotting, and β-actin was used as a loading control. (**B**) Blots were quantified using ImageJ™ software and presented. Data are shown as the mean ± standard deviation of samples and p-value represents statistical significance ($p^* < 0.05$; $p^{**} < 0.01$; $p^{***} < 0.001$ and $p^{****} < 0.0001$) and the data was analysed using Weltch's t test.
